# Surgical Management of Giant L2 Adrenal Neuroblastoma in Adult Male

**DOI:** 10.1155/2020/8890223

**Published:** 2020-12-05

**Authors:** Bikash Bikram Thapa, Sanjay Yadav, Sujit Pant, Pratik Rajkarnikar, Pankaj Mandal

**Affiliations:** ^1^Department of Surgery, College of Medicine, Nepalese Army Institute of Health Sciences, Nepal; ^2^Department of Radio Diagnosis, College of Medicine, Nepalese Army Institute of Health Sciences, Nepal; ^3^Department of Pathology, College of Medicine, Nepalese Army Institute of Health Sciences, Nepal

## Abstract

Neuroblastoma is an embryonal malignancy that arises from neural crest cells. Adult adrenal neuroblastoma is a rare disease, and less than 100 cases were reported in the literature. Adult neuroblastoma commonly presents with abdominal (retroperitoneal) lump and pain. A 35-year-old male patient presented with a giant (20 cm × 17 cm × 12 cm) nonfunctional left adrenal mass. He underwent en-bloc surgical excision of the left adrenal gland along with the left kidney. Histopathological examination revealed adrenal neuroblastoma (stage 2B, L2). We present here the surgical management of the rare adult adrenal neuroblastoma.

## 1. Introduction

Neuroblastoma (NB) is the most common extracranial solid malignancy in children that account for 15% of the cancer mortality [1]. Median age at diagnosis is 17 months, and only 2% of cases are found in patients above 10 years [2]. Less than 100 cases of adult neuroblastoma were reported in literature since it was first reported on 1957 by a Dutch physician [3–5]. Patients usually present with abdominal mass, pain, and other constitutional symptoms like fever, high blood pressure, flushing, and paraneoplastic syndrome [6]. There is no standard treatment protocol for adult neuroblastoma. Adults have lower tolerance to the treatment with low five-year overall survival rate (36% in adult vs 85% in infant) [7].

## 2. Case Report

A 35-year-old male with history of neurocysticercosis under medication presented to the surgery department with left upper abdominal pain and lump of one-week duration. The pain was acute in onset, dull aching type, intermittent, and severe enough to disturb daily activities. He was non-smoker, non-alcoholic, normotensive, and non-diabetic with no family history of malignancy. His body mass index was 25.3 kg/m^2^. Clinical examination revealed a palpable mass in the left hypochondrium extending 7 cm below the left costal margin that was firm, smooth, and nontender.

**Figure 1:**
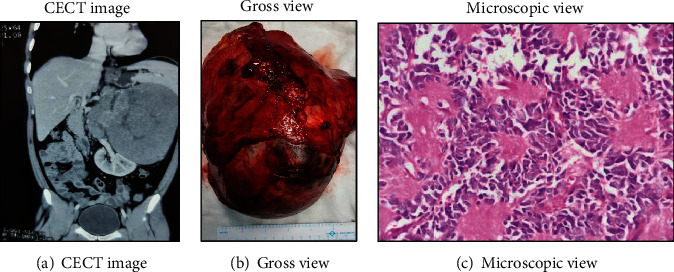
Left adrenal neuroblastoma.

The computed tomography with contrast (CECT) of the chest and abdomen-pelvis (Figure 1(a)) showed a well-defined multilobulated mass lesion measuring 16 × 13 × 16.5 cm with heterogeneous enhancement. The left suprarenal gland was not visualized separately. The mass lesion was abutting and displacing the pancreas, spleen, and left kidney. The lobulated mass was extending across the midline abutting and displacing the abdominal aorta, renal vessels, and encasing the left renal artery (Figure 1(a)). The complete blood count, renal function test, and liver function test were within the normal limit. The serum and blood metanephrines were in the normal limit; 36.1 pg/ml and 74.29 *μ*g/day, respectively. Serum cortisol and aldosterone were normal. Patient was managed with en-bloc left adrenalectomy, left nephrectomy, and paraortic lymph node dissection through left subcostal hockey stick incision under epidural-general anesthesia. The left renal vessels were completely encased, and abdominal aorta was partially encased by the lymph nodal mass. The surgery last for 210 minutes with blood loss of approximately 300 ml. The patient was discharged on postoperative day 7. Gross histopathology examination of the surgical specimen (Figure 1(b)) revealed adrenal mass of size 20 × 17 × 12 cm. Microscopic examination showed small round blue tumor cells arranged around the central area filled with neurofibrillary processes giving rosette appearance—Homer-Wright rosettes (Figure 1(c)). Tumor cells were differentiating with low mitotic index, and the margins were negative. The hilar lymph node (6/6) was positive for malignancy. Kidney margins were negative for malignancy. We could not perform Metaiodobenzylguanidine (MIBG)scan postoperatively due to lack of facility inside the country. However, the technetium bone scan was negative for bone metastasis. Patent was counseled by a clinical oncologist regarding adjuvant therapy but choose to continue follow-up. The CECT scan done 6 months postsurgery was normal.

## 3. Discussion

Neuroblastoma consists of a group of neuroblastic tumors (neuroblastomas, ganglineuroblastomas, and ganlioneuromas) that originate from neural crest cells. It is exclusively the disease of childhood. The incidence rate is 60% before the age of one year drops to 10% at age of 14 years. The incidence rate for patients aged over 30 years is 0.2 cases per million inhabitants per year [8]. Out of the 112 adult neuroblastoma patients reported from M.D. Anderson cancer center in 2014, there were 48% L1, 27% L2, and 14% M diseases [9].

The clinical presentation of neuroblastoma depends on the tumor's location, local invasion, and metastasis. The head and neck region, central nervous system, chest cavity, and abdominal cavity are most common primary sites of adult neuroblastoma. About 50% of the abdominal neuroblastoma is adrenal in origin [4, 9, 10]. Retroperitoneal neuroblastoma of size as big as 25 × 12 × 18 cm was reported in literature [11]. Our patient had stage 2B adrenal neuroblastoma based on the international neuroblastoma staging system (1990) [12]. According to the international neuroblastoma risk group staging system [13], he had L2M0 disease (locoregional disease involving the left renal pedicle with no metastasis).

Management of neuroblastoma requires a multimodality approach that includes surgery, radiotherapy, high-dose chemotherapy with stem cell rescue, and Iodine131-MIBG therapy [5, 9]. A retrospective study on stage I and II adult neuroblastoma (age group > 12 years) showed 83% 5-year overall survival (OS) after multimodality treatment. Surgery remains the choice of treatment for the local control of the disease [5]. We performed open transperitoneal adrenalectomy because of the size of the tumor and extent of the local spread. Laparoscopic adrenalectomy is gaining wider popularity over open adrenalectomy (then gold standard) since early 90s. A Cochrane review published on 2018 found no difference on overall early and late complications between intraperitoneal and retroperitoneal laparoscopic adrenal surgery [14].

Guidelines suggest laparoscopic adrenalectomy for malignant adrenal tumor with size less than 6 cm [15]. Number of studies had cautioned about the poor oncological outcome of the laparoscopy adrenal surgeries. However a meta-analysis reported a comparable outcome between the open and laparoscopic approaches in the management of adrenal malignancy [16, 17]. Laparoscopic excision of adrenal neuroblastoma (5 to 8 cm) has been successfully performed in recent years [4, 18]. The robot-assisted laparoscopic approach has added advantages over the laparoscopic approach for the management of the adrenal tumors due to its three-dimensional magnified vision and dexterity [19].

There is lack of standard treatment guidelines in the adult population due to rarity of the disease. The Surveillance, Epidemiology, and End-Results registry (SEER) database had a total of 181 adult neuroblastomas from 1973 to 2010. In those cohort of 181 adult (18-60 years) neuroblastomas, L1 and L2 diseases had better progression-free survival (PFS) and overall survival (OS) with the combination of surgery and radiation than surgery alone [8]. Adjuvant chemotherapy had no added benefit in progression-free survival (PFS) in adult L1 and L2 neuroblastomas [9]. Patient age at diagnosis, stage, histology, tumor grade, MYCN oncogene status, chromosome 11q status, and DNA ploidy are important prognostic factors for neuroblastoma [5, 9]. In recent days, attention has been given to miRNA (microRNA) and its relationship with the molecular pathogenesis of neuroblastoma that has important implications in the diagnosis, prognosis, and miRNA-based therapy for neuroblastoma [20].

## 4. Conclusion

Adult adrenal neuroblastoma of this size is the first of its kind in literature. Surgical management in L2 adrenal neuroblastoma is advised for local control of the disease. Minimal invasive surgery is a new standard for adrenal tumor. Multimodality treatment can offer long-term favorable oncological outcome in adult neuroblastoma. The treatment outcome is multifactorial. Larger and longer study series of the adult neuroblastoma can guide us through the challenges posed by the rarity of the disease.

## Data Availability

Case-related information is all described.
